# Reliability of point-of-care shoulder ultrasound measurements for subacromial impingement in asymptomatic participants

**DOI:** 10.3389/fresc.2022.964613

**Published:** 2022-08-17

**Authors:** Xiaoning Yuan, Ryan Lowder, Kathelynn Aviles-Wetherell, Christian Skroce, Katherine V. Yao, Jennifer Soo Hoo

**Affiliations:** ^1^ Department of Rehabilitation Medicine, Weill Cornell Medicine, New York, NY, United States; ^2^ Department of Physical Medicine and Rehabilitation, Musculoskeletal Injury Rehabilitation Research for Operational Readiness (MIRROR), Uniformed Services University of the Health Sciences, Bethesda, MD, United States

**Keywords:** musculoskeletal ultrasound, rehabilitation, reliability, shoulder impingement, shoulder pain

## Abstract

**Background:**

Rehabilitation is the key to management of patients with subacromial impingement syndrome to prevent disability and loss of function. While point-of-care musculoskeletal ultrasound aids clinical diagnosis of subacromial impingement syndrome, many patients do not demonstrate the classic findings of dynamic supraspinatus tendon impingement beneath the acromion on ultrasound. The objective of this study was to establish the most reliable shoulder ultrasound measurements for subacromial impingement, by evaluating the intra-rater and inter-rater reliability of measurements in asymptomatic participants.

**Methods:**

Eighteen participants (9 women, 9 men, mean ± standard deviation: 34.6 ± 7.9 years of age) underwent bilateral shoulder ultrasound evaluations with measurements for subacromial impingement (acromiohumeral distance, acromion-greater tuberosity distance, supraspinatus tendon, subacromial-subdeltoid bursa, and subacromial-subdeltoid bursal fluid thickness) performed by two sports medicine physicians. Intra-class coefficients were calculated to determine the intra- and inter-rater reliability of shoulder ultrasound images and measurements.

**Results:**

Intra-rater reliability for acromiohumeral distance (0.76–0.79), supraspinatus tendon (0.91–0.95), subacromial-subdeltoid bursa (0.76–0.84), and subacromial-subdeltoid bursal fluid thickness (0.75–0.81) was found to be good to excellent, whereas inter-rater reliability ranged from poor to moderate.

**Conclusions:**

Acromiohumeral distance in neutral position and short axis ultrasound measurements of supraspinatus tendon, subacromial-subdeltoid bursa, and subacromial-subdeltoid bursal fluid thickness in the modified Crass position were the most reliable for subacromial impingement in asymptomatic participants. We recommend validation of these measurements in a symptomatic population to aid diagnosis and direct rehabilitation of patients with suspected subacromial impingement, and to increase point-of-care ultrasound uptake, availability, and training among rehabilitation professionals across health systems.

## Introduction

Subacromial impingement syndrome (SIS) accounts for 44%–65% of all shoulder complaints and disorders in patients presenting to clinic (1). A challenge to the clinical diagnosis of subacromial impingement is its variability in presentation, as multiple structural and biomechanical abnormalities can contribute to symptoms, coupled with a lack of consensus on diagnostic criteria (2). The utility of point-of-care musculoskeletal ultrasound (MSK US) in rehabilitation settings can aid in the diagnosis of shoulder impingement and exclude other causes of shoulder pain (3). The classic finding of subacromial impingement using US is demonstration of dynamic supraspinatus tendon (SST) or subacromial-subdeltoid bursa (SASDB) bunching beneath the acromion (4). However, despite the advances and expanded use of MSK US in rehabilitation clinics, most patients diagnosed with clinical SIS do not have either of these findings on US. To date, no other standardized, objective US findings are widely utilized in clinical practice, although it is generally accepted that acromiohumeral distance (AHD), SST thickness, SASDB thickness, and SASDB fluid thickness likely correspond with SIS symptoms (5, 6).

AHD was originally described for radiographs as the shortest distance between the inferior aspect of the acromion and the humeral head, with RTC abnormalities associated with reductions in AHD secondary to superior migration of the humeral head (7). AHD is by far the most frequently studied shoulder US measurement in reliability studies of asymptomatic (8–13) and symptomatic (14–22) participants, with reported reliability ranging from poor to excellent intra-rater reliability (8–12, 19–22), and moderate to excellent inter-rater reliability (13–18). AGT (acromion-greater tuberosity) distance is the shortest distance between the inferolateral edge of the acromion and the apex of the greater tuberosity and has proven to be useful for examining patients with impingement symptoms (23–25). Past studies reported good to excellent intra-rater reliability (26, 27), and moderate to good inter-rater reliability (26, 28) of AGT distance measurements by US.

Thickening of the SST and SASDB in the setting of overuse can lead to reduced subacromial space and subsequent development of SIS (29). Increased SST thickness has been reported in patients with SIS and RTC tendinopathy, compared to asymptomatic patients (20, 30, 31), although Cholewinski et al. (2008) reported conflicting evidence of decreased SST thickness in SIS patients (3). The intra-rater and inter-rater reliability of US measurements of SST thickness has ranged from good to excellent in prior studies of asymptomatic (10, 32) and SIS patients (14, 15, 17, 20). Two studies of US measurements of SASDB thickness in asymptomatic (32) and SIS (14) participants reported good to excellent intra-rater and inter-rater reliability. However, to date, no published studies have examined reliability of US measurements of SASDB fluid thickness in symptomatic or asymptomatic populations.

Prior studies examining the inter-rater reliability of image interpretation are limited to AHD measurement only, reporting only moderate to good reliability in asymptomatic or symptomatic participants (8, 13, 16). The inter-rater reliability of image interpretation for other shoulder US measurements relevant to SIS has not been published to date.

The objectives of the present study were threefold: (1) to describe the inter-rater and intra-rater reliability of US imaging and measurements of the AHD, AGT distance, SST, and SASDB thickness in asymptomatic volunteers; (2) to evaluate the inter-rater and intra-rater reliability of US imaging and measurement of the SASDB fluid thickness in asymptomatic volunteers; and (3) to assess the reliability of inter-rater image interpretation of all shoulder US measurements in asymptomatic participants. To the authors' knowledge, this study is the first to establish the most reliable among 11 distinct shoulder US measurements for evaluation of subacromial impingement. The data obtained from this comprehensive study are necessary to aid diagnosis and direct rehabilitation of patients with shoulder disorders, to increase point-of-care US uptake, availability, and training among rehabilitation professionals, and to support future clinical studies comparing symptomatic and asymptomatic patient populations.

## Materials and methods

### Study sample

Study participants were enrolled in this study from February to June 2020 at a large urban academic medical center. The study protocol was approved by the local Institutional Review Board (Protocol # 19-08020707). Inclusion criteria for enrollment were asymptomatic male or female participants between 20–50 years old who were able to provide informed consent. Participants were excluded if they had shoulder pain during or within six months of their date of study participation, received a corticosteroid injection or had taken oral corticosteroids within six months of their study participation, or had any history of shoulder surgery, rotator cuff tear, or shoulder dislocation, based upon self-report. Following informed consent, all participants completed a demographics and medical history survey.

### Shoulder US protocols

US evaluations were performed by two sonographers (Rater A and Rater B) who are Physical Medicine and Rehabilitation physicians with fellowship training in Sports Medicine with at least three years of experience performing diagnostic musculoskeletal US examinations. All US evaluations were conducted using SonoSite X-Porte US systems with 6–15 MHz linear transducers. Three training sessions were completed prior to study enrollment by both sonographers to review US methodology.

Each participant underwent three bilateral shoulder US evaluations, two by Rater A and one by Rater B. Rater A's first and second US evaluations were at least 15 min apart. For each evaluation, the sonographer acquired US images that were used to obtain 11 quantitative measurements for both shoulders: acromiohumeral distance (AHD) (in neutral and at 60° active shoulder abduction), acromion-greater tuberosity (AGT) distance, supraspinatus tendon (SST) thickness (long and short axis), subacromial-subdeltoid bursa (SASDB) thickness (long axis #1 and #2, short axis), and SASDB fluid (long axis #1 and #2, short axis). For all measurements, the participant was seated in a chair with feet flat on the ground.

**Figure 1:**
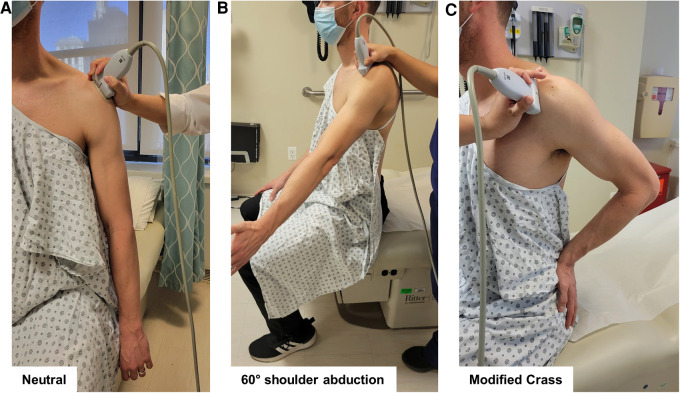
Participant positioning and transducer placement for shoulder ultrasound (US) evaluations. To measure acromioclavicular distance (AHD), each arm was examined in two positions: (**A**) in neutral position with the forearm pronated, resting on the ipsilateral thigh, and (**B**) at 60° active shoulder abduction in the coronal plane, as verified by goniometry. The US transducer is positioned in the coronal plane, parallel to the longitudinal axis of the humerus. To measure AGT distance, each arm was examined in neutral position with the forearm pronated, as described for AHD (**A**). To measure SST, SASDB, and SASDB fluid thickness, the participant was positioned with the palm of the examined arm resting on the posterior iliac crest and the elbow directed posteriorly (modified Crass position; (**C**). US transducer placement depicts positioning to obtain long axis views of the SST, SASDB, and SASDB fluid.

**Figure 2:**
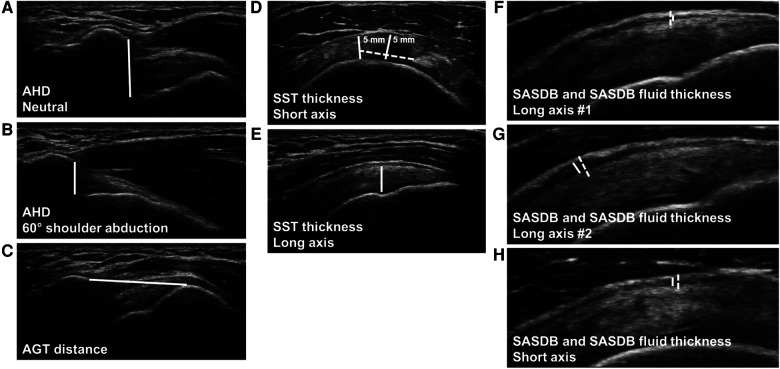
Ultrasound (US) measurements of acromiohumeral distance (AHD), acromion-greater tuberosity (AGT) distance, supraspinatus tendon (SST) thickness, and subacromial-subdeltoid bursa (SASDB) and SASDB fluid thickness. US images were captured in two positions for AHD measurements: (**A**) in neutral position with the forearm pronated, resting on the ipsilateral thigh, and (**B**) at 60° active shoulder abduction in the coronal plane. AHD was measured as the shortest tangential distance (line) between the hyperechoic landmarks of the acromion and the superior-most aspect of the humerus. (**C**) AGT distance was measured as the shortest distance (line) between the lateral edge of the acromion and the apex of the greater tuberosity of the humerus. SST thickness in short axis (**D**) was obtained as the average of measurements (lines) at two points, 5 mm and 10 mm posterior to the edge of the biceps tendon (dashed lines). SST thickness in long axis (**E**) was measured at the deepest portion of the superior facet of the greater tuberosity (line). SASDB thickness (dashed lines) was measured by obtaining images in three positions: in the longitudinal plane over the more objective, anterior-most portion of the greater tuberosity (long axis #1, **F**) and the subjective point of greatest thickness, determined by the rater (long axis #2, **G**), and in the transverse plane (short axis, **H**). SASDB fluid (lines) was measured as a hypoechoic line between two layers of peribursal fat in the same three positions (long axis #1 and #2, short axis, **F–H**).

To measure AHD, each arm was examined in two positions, as previously described: (1) in neutral position with the forearm pronated, resting on the ipsilateral thigh (Figure 1A), and (2) at 60° active shoulder abduction in the coronal plane, as verified by goniometry (Figure 1B) (8, 33). The US transducer was positioned in the coronal plane, parallel to the longitudinal axis of the humerus, to visualize the shortest tangential distance between the hyperechoic landmarks of the acromion and the superior-most aspect of the humerus (Figures 2A,B) (5).

To measure AGT distance, each arm was examined in neutral position with the forearm pronated, as described for AHD. The US transducer was positioned to visualize the shortest distance between the lateral edge of the acromion and the apex of the greater tuberosity of the humerus (Figure 2C) (3, 25).

To measure SST thickness, the participant was positioned with the palm of the examined arm resting on the posterior iliac crest and the elbow directed posteriorly (modified Crass position) (Figure 1C). The US transducer was positioned on the shoulder to view the SST in short axis, translating anteriorly to the rotator interval, until the intra-articular portion of the long head of the biceps tendon was visualized. SST thickness in short axis was obtained as the average of measurements at two points, 5 mm and 10 mm posterior to the edge of the biceps tendon (Figure 2D). The transducer was then repositioned to view the SST in long axis over the anterior-most portion of the greater tuberosity. SST thickness in long axis was measured at the deepest portion of the superior facet of the greater tuberosity (Figure 2E) (34).

To measure SASDB and SASDB fluid thickness, the participant's arm was again positioned in modified Crass. The US transducer was positioned perpendicular to the cortex of the humeral head to avoid obliquity of the bursa, which appears as a line between the overlying deltoid muscle and the underlying SST in long axis. SASDB thickness, encompassing the superficial peribursal fat, bursal fluid, and deep peribursal fat, was measured by obtaining images in three positions: in the longitudinal plane over the more objective, anterior-most portion of the greater tuberosity (long axis #1, Figure 2F) and the subjective point of greatest thickness, determined by the rater (long axis #2, Figure 2G), and in the transverse plane (short axis, Figure 2H) (6, 24). Measurements of the SASDB fluid, a hypoechoic line between two layers of peribursal fat, were obtained in the same three positions (long axis #1 and #2, short axis, Figures 2F–H), using the SASDB images from each respective view (6, 24).

Each rater performed measurements of their saved images, following acquisition of all images per participant. In addition, Rater A obtained measurements from images taken during Rater B's US evaluation to assess the inter-rater reliability of image interpretation, given the same images. Sonographers were blinded to each other's measurements.

### Statistical analysis

All statistical analyses were performed using SPSS Statistics 25 (IBM Corp., Armonk, NY), Excel for Microsoft 365 (Redmond, WA), and GraphPad Prism 7.05 (GraphPad Software, San Diego, CA).

All data were first assessed for normality using the Shapiro-Wilk test. Inter-rater and intra-rater reliability of US evaluations were assessed by calculating intra-class correlation coefficients (ICC) with 95% confidence intervals (CI). The measurements obtained by Rater A's US evaluations were used to assess intra-rater reliability (two-way mixed effects model, absolute agreement, single measures), and those obtained by Raters A and B were used to assess inter-rater reliability (two-way random effects model, absolute agreement, single measures). ICC values greater than 0.9 (inclusive) were considered to be excellent reliability, values between 0.75 (inclusive) and 0.90 to be good reliability, values between 0.5 (inclusive) and 0.75 to be moderate reliability, and values below 0.5 to be poor reliability (35). Minimal detectable differences (MDDs) were also calculated for the US measurements, representing the minimal difference necessary between measurements that do not result from random variation or measurement error (17).

Bland-Altman analysis was performed to detect systematic bias, by comparing differences between Raters A and B's US images and measurements per participant against their means, with 95% limits of agreement (LOA) (36).

## Results

### Study population

Eighteen participants (9 women, 9 men) ranging from 23 to 49 years of age completed this study. None of the participants reported history of chronic inflammation, diabetes, osteoarthritis, rheumatoid arthritis, reactive arthritis, obesity, fibromyalgia, or gout, and their current jobs did not involve repetitive overhead lifting. Additional demographic parameters are listed in Table 1.

**Table 1:** Demographics of participants (*n* = 18).

		*n*	%	Mean ± SD
Age (years)				34.6 ± 7.9
BMI (kg/m^2^)				23.4 ± 2.6
Sex	Male	9	50	
	Female	9	50	
Dominant side	Right	17	94.4	
	Left	1	5.6	
Job involves overhead lifting	N/A	7	38.9	
	0%–25%	11	61.1	
Exercise with upper body weights	N/A	4	22.2	
	1–2 times per week	11	61.1	
	3–4 times per week	2	11.1	
	5–6 times per week	1	5.6	
History of shoulder pain limiting ADLs (greater than six months prior to study participation)	Yes	3	16.7	
	No	15	83.3	
Current NSAID use^a^	Yes	3	17.6	
	No	14	82.4	

ADLs, activities of daily living; BMI, body mass index; N/A, not applicable; NSAID, non-steroidal anti-inflammatory drug.

^a^ Out of 17 participant questionnaire responses.

### Quantitative shoulder US measurements for asymptomatic participants

One participant was excluded from US measurement analyses due to missing US images caused by a technical error. The mean and standard deviation of US measurements from Rater A and B's images are reported in **Supplementary Table S1**.

### Reliability

Inter- and intra-rater reliability values are reported in Table 2. Inter-rater reliability of image interpretation (comparing Rater A's measurements of Rater B's US images to Rater B measurements of the same images) are reported in the third column of Table 2. Inter-rater reliability ranged from poor for SASDB fluid thickness in long axis #1 to moderate for SST thickness in short axis. Intra-rater reliability ranged from moderate for AGT distance to excellent for SST thickness in long axis. The majority of ICCs for inter-rater reliability were within the moderate range, whereas the majority of ICCs for intra-rater reliability fell within the good range. For inter-rater reliability of image interpretation, the majority of ICCs were excellent, ranging from good for SASDB thickness in long axis #2 to excellent for AHD.

**Table 2:** Intra-class correlation coefficients for inter-rater and intra-rater reliability.

		Ultrasound measurements						Image interpretation		
		Inter-rater reliability			Intra-rater reliability			Inter-rater reliability		
		ICC	95% CI	MDD (cm)	ICC	95% CI	MDD (cm)	ICC	95% CI	MDD (cm)
AHD	Neutral	0.63	0.37–0.80	0.33	0.79	0.62–0.89	0.20	0.96	0.92–0.98	0.04
AHD	60° abduction	0.57	0.29–0.76	0.36	0.76	0.57–0.87	0.17	0.98	0.96–0.99	0.02
AGT distance		0.58	0.30–0.77	0.78	0.68	0.45–0.83	0.55	0.96	0.92–0.98	0.08
SST thickness	Long axis	0.60	0.34–0.78	0.11	0.95	0.90–0.97	0.01	0.86	0.75–0.93	0.04
	Short axis	0.64	0.38–0.80	0.11	0.91	0.83–0.96	0.02	0.92	0.83–0.96	0.02
SASDB thickness	Long axis #1	0.49	0.20–0.71	0.08	0.84	0.71–0.92	0.02	0.88	0.51–0.96	0.02
	Long axis #2	0.56	0.13–0.66	0.08	0.76	0.55–0.87	0.03	0.79	0.64–0.89	0.04
	Short axis	0.54	0.29–0.75	0.08	0.76	0.56–0.87	0.03	0.92	0.60–0.89	0.02
SASDB fluid thickness	Long axis #1	0.43	0.31–0.76	0.05	0.75	0.50–0.91	0.02	0.80	0.78–0.95	0.02
	Long axis #2	0.58	0.23–0.75	0.04	0.80	0.57–0.87	0.02	0.90	0.85–0.96	0.01
	Short axis	0.53	0.25–0.73	0.05	0.81	0.65–0.90	0.02	0.92	0.84–0.96	0.01

AGT, acromion-greater tuberosity; AHD, acromiohumeral distance; CI, confidence interval; ICC, intra-class correlation coefficient; MDD: minimal detectable difference; SASDB, subacromial-subdeltoid bursa; SST, supraspinatus tendon.

The MDDs were consistently lower for intra-rater compared to inter-rater reliability across all shoulder US measurements (Table 2). The greatest MDDs were calculated for AGT distance across intra- and inter-rater reliability. MDDs for inter-rater image interpretation were low overall, with the exception of AGT distance.

Bland-Altman analyses including calculation of bias and 95% limits of agreement (LOA) are reported in **Supplementary Table S2**. No systematic bias was detected, compared to clinically relevant cut-off values for each US measurement. Bland-Altman plots depicting differences in measurements of US images between raters as a function of their means are depicted in **Supplementary Figure S1**.

## Discussion

Rehabilitation is the key to management of patients with SIS to prevent disability and loss of function. The purpose of this study was to evaluate the reliability of 11 distinct US measurements relevant to subacromial impingement in asymptomatic participants. Intra-rater reliability for the majority of US measurements was found to be good to excellent, with the exception of AGT distance (moderate), whereas inter-rater reliability ranged from poor to moderate. No systematic bias was detected by Bland-Altman analyses for the shoulder US measurements performed in this study.

Of the shoulder US measurements assessed in this study, AHD reliability is the most frequently studied (8–12, 14, 19–22). In the present study, we found that the intra-rater reliability for AHD in both the neutral and 60° abduction positions was good, which generally aligns with previous research that demonstrated good to excellent intra-rater reliability for AHD in both positions (9–12, 14, 17, 19–22). Prior studies of the inter-rater reliability of AHD measurements in asymptomatic participants were less conclusive, ranging from moderate, good (8, 9), to excellent (14, 18). In the present study, the inter-rater reliability for both AHD measurements was found to be moderate. Notably, in the studies reporting excellent inter-rater reliability, preliminary data collection, training and overall agreement phases, requiring at least 80% agreement, were completed prior to formal data collection for reliability studies (14, 18). Given the variability in inter-rater reliability of AHD measurements reported previously and in the current study, we agree with the authors of a systematic review of intra- and inter-rater reliability of radiological methods of AHD measurements, who concluded that AHD measurement using US is presently most reliable for a single operator (37).

We found moderate intra- and inter-rater reliability of US imaging and measurement of AGT distance in asymptomatic participants (34.6 ± 7.9 years), in comparison to prior studies that reported good to excellent intra-rater reliability and moderate to good inter-rater reliability in asymptomatic individuals. Decreased variability in shoulder anatomy among asymptomatic participants of a narrower age range (mean ± SD: 21 ± 2 years (26); 64 ± 10.5 years (27); 54 ± 5 years (28) may account for the higher reliability reported in studies by Kumar et al. (26–28), compared to the present study (mean ± SD: 34.6 ± 7.9 years). At this time, we do not recommend using AGT distance as an outcome measure in future studies, until further refinement of US protocol can be made to ensure improved reliability across all age groups.

The intra-rater reliability of SST thickness found in the present study was excellent, which corresponds with prior studies that also reported excellent intra-rater reliability (10, 14, 20, 32). However, our inter-rater reliability was only moderate, in comparison to one prior study that reported good to excellent inter-rater reliability of US measurements of SST thickness obtained in different shoulder positions (14) than the modified Crass used in the present study. Two studies previously reported good to excellent inter- and intra-rater reliability of US measurement of SASDB thickness in asymptomatic participants (14, 32), using different shoulder positioning. In the present study, US measurements of SASDB thickness were obtained from shoulders in the modified Crass position by clinical convention (38), and demonstrated good intra-rater reliability and poor to moderate inter-rater reliability.

To our knowledge, no previous studies have assessed the inter- or intra-rater reliability of SASDB fluid thickness in asymptomatic participants. We report good intra-rater reliability of SASDB fluid thickness in long and short axis, and poor to moderate inter-rater reliability, respectively. Two different long axis views were evaluated for US measurements of SASDB and bursal fluid to compare their reliability. The first method for visualizing the SASDB and bursal fluid in long axis was more objective, using a defined reference point each time, at the anterior-most portion of the greater tuberosity, which we expected to yield higher reliability. The second method was more subjective, but more clinically relevant, wherein the sonographer identifies the area of greatest SASDB and bursal fluid thickness, which we expected to be more clinically relevant. Surprisingly, we found that the inter-rater reliability was lower using the first method (long axis #1, more objective) than the second (long axis #2, more subjective), when evaluating SASDB and bursal fluid thickness. While the intra-rater reliability of SASDB fluid thickness was higher in long axis #2 (subjective) than #1 (objective), the intra-rater reliability of SASDB thickness was higher in long axis #1 (objective). Overall, our findings demonstrated that the more objective method for obtaining US images and measurements was not consistently superior to the more subjective but more clinically relevant method. If two methods for imaging and measuring a shoulder structure are found to be of similar reliability during research studies, the more clinically relevant method may be favored for use in clinical practice, even if there is more room for subjectivity.

Overall, our study demonstrated better intra- than inter-rater reliability of US shoulder measurements relevant to subacromial impingement. This finding corresponds with the conclusion of a systematic review of the reliability of diagnostic imaging for measuring tendon size, including thickness, across studies of all tendon sites, which reported combined inter-rater reliability ranging from poor to excellent, and intra-rater reliability from moderate to excellent (39). Based on the present findings and the overall literature, we recommend that US measurement of AHD in neutral position, and short axis US measurements of SST, SASDB, and SASDB fluid thickness in the modified Crass position be utilized in future research. AHD in neutral position yielded among the highest inter-rater reliability of images and measurements and can serve as a reference measurement to prior US reliability studies. Inter-rater reliability of short axis measurements for SST, SASDB, and SASDB fluid thickness were typically higher than their long axis correlates in our study.

Few studies have reported inter-rater reliability for shoulder US image interpretation, in which different raters measure the same set of images. Our study yielded good to excellent inter-rater reliability for image interpretation, in comparison to previous studies that demonstrated only moderate to good reliability for asymptomatic or symptomatic participants (8, 13, 16). The inter-rater reliability of US measurements that we advocate for use in future studies, AHD in neutral position, and SST, SASDB, and SASDB fluid thickness in short axis, all fell within the excellent range for image interpretation. In contrast, the inter-rater reliability of different raters capturing images and performing these measurements were only in the moderate range, which suggests that differences in reliability are operator-dependent, resulting from differences in probe positioning to obtain images rather than differences in image interpretation. As the intra-rater MDDs across all shoulder US measurements were lower than their respective inter-rater values, it seems overall advantageous for a single operator to capture US images and perform measurements when possible.

Limitations of the present study include the small sample size of 17 participants with available US measurements for analysis. Past reliability studies of shoulder US ranged in recruitment from 10 to 11 to upwards of 83 participants (8–10, 15, 28, 32). Although three training sessions were completed by both sonographers, extended training requiring at least 80% agreement between sonographers before proceeding to the formal study is likely to improve the inter-rater reliability of shoulder US measurements, but may not be feasible in clinical practice. Finally, all US evaluations in the present study were performed on the same day, at least 15 min apart, to minimize potential physiologic changes in bursal fluid volume over a longer time period. Follow-up studies may consider repeating US evaluations of the cohort on a different day to allow for assessment of test-retest reliability for shoulder US measurements.

The results of this study can inform future US studies of patients with shoulder pain concerning for subacromial impingement. While we report novel findings for reliability of US measurements of SASDB fluid thickness in this study of asymptomatic participants, further studies are necessary to validate the diagnostic utility and reliability of this and other recommended US measurements in patients with shoulder pain and suspected subacromial impingement. Past research observed that the reliability of utilizing US to measure shoulder structures is higher in SIS patients than in asymptomatic participants (14). Indeed, the inter-rater reliability of US measurements in asymptomatic participants in the present study are lower than inter-rater reliability reported in studies of SIS patients (14, 15). Therefore, we may expect better reliability in future studies of patients with SIS, who may demonstrate greater sonographic evidence of SST and SASDB thickening or inflammation. Ultimately, established US approaches to evaluation and monitoring of patients with shoulder disorders can guide rehabilitation of patients with suspected subacromial impingement, and increase point-of-care ultrasound uptake, availability, and training among rehabilitation professionals across health systems.

## Data Availability

The raw data supporting the conclusions of this article will be made available by the authors, without undue reservation.
