# Supplementary material for: Reliability of point-of-care shoulder ultrasound measurements for subacromial impingement in asymptomatic participants
Source: Front Rehabil Sci. 2022 Aug 17;3:964613. doi: 10.3389/fresc.2022.964613 (PMC9397902; doi:10.3389/fresc.2022.964613)
Supplement: Supplementary file 1 [file Table_1_v1.docx]

Supplementary Material

# Supplementary Data

N/A

# Supplementary Figures and Tables

**Supplementary Table 1.** Shoulder ultrasound measurements for a healthy control population. Rater A performed two evaluations, and Rater B performed one evaluation. Thirty-four measurements were obtained per evaluation (*n* = 34 for bilateral shoulder ultrasound evaluations of 17 participants). All measurements are reported as mean ± standard deviation in cm.

| Measurements | | Rater A | | Rater B |
| --- | --- | --- | --- | --- |
|  | | Evaluation #1 | Evaluation #2 |  |
| AHD | Neutral | 1.08 ± 0.24 | 1.12 ± 0.24 | 1.07 ± 0.21 |
| AHD | 60° abduction | 0.74 ± 0.18 | 0.71 ± 0.18 | 0.69 ± 0.24 |
| AGT distance |  | 1.90 ± 0.41 | 1.89 ± 0.46 | 1.94 ± 0.52 |
| SST  thickness | Long axis | 0.44 ± 0.07 | 0.44 ± 0.07 | 0.45 ± 0.06 |
|  | Short axis | 0.42 ± 0.07 | 0.41 ± 0.07 | 0.41 ± 0.08 |
| SASDB  thickness | Long axis #1 | 0.17 ± 0.04 | 0.17 ± 0.04 | 0.18 ± 0.05 |
|  | Long axis #2 | 0.20 ± 0.04 | 0.21 ± 0.04 | 0.22 ± 0.06 |
|  | Short axis | 0.21 ± 0.04 | 0.20 ± 0.03 | 0.23 ± 0.06 |
| SASDB fluid  thickness | Long axis #1 | 0.05 ± 0.02 | 0.05 ± 0.02 | 0.04 ± 0.02 |
|  | Long axis #2 | 0.07 ± 0.03 | 0.08 ± 0.03 | 0.07 ± 0.03 |
|  | Short axis | 0.08 ± 0.03 | 0.08 ± 0.03 | 0.07 ± 0.03 |

AGT = acromion-greater tuberosity; AHD = acromiohumeral distance; SASDB = subacromial-subdeltoid bursa; SST = supraspinatus tendon.

**Supplementary Table 2.** Bland-Altman analysis for the inter-rater reliability of ultrasound images and measurements.

|  |  | Bias (cm) | 95% LOA (cm) |
| --- | --- | --- | --- |
| AHD | Neutral | 0.02 | -0.37–0.40 |
| AHD | 60° abduction | 0.05 | -0.37–0.48 |
| AGT distance |  | -0.04 | -0.89–0.81 |
| SST thickness | Long axis | -0.01 | -0.13–0.11 |
|  | Short axis | 0.00 | -0.12–0.13 |
| SASDB thickness | Long axis #1 | -0.01 | -0.09–0.07 |
|  | Long axis #2 | 0.01 | -0.04–0.05 |
|  | Short axis | -0.01 | -0.10–0.08 |
| SASDB fluid thickness | Long axis #1 | 0.01 | -0.04–0.05 |
|  | Long axis #2 | -0.02 | -0.11–0.07 |
|  | Short axis | 0.01 | -0.05–0.06 |

AGT = acromion-greater tuberosity; AHD = acromiohumeral distance; CI = confidence interval;

LOA = limits of agreement; SASDB = subacromial-subdeltoid bursa; SST = supraspinatus tendon.

## Supplementary Figures


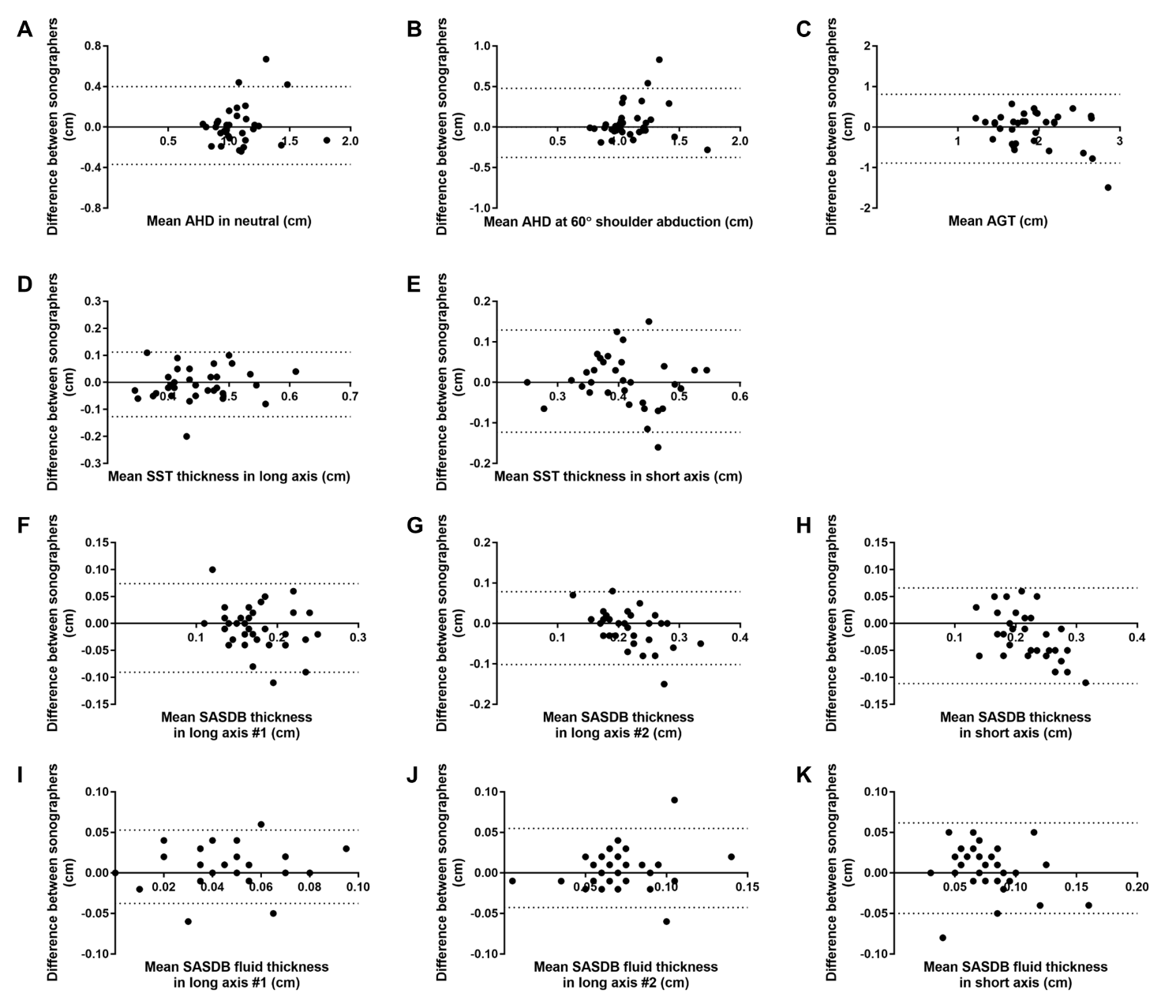


**Supplementary Figure 1.** Bland-Altman plots depicting inter-rater reliability, showing the mean difference between the two sonographers when imaging and measuring (**A**) acromiohumeral distance (AHD) in neutral position (ICC = 0.63; bias = 0.02 cm; 95% LOA = -0.37–0.40 cm); (**B**) AHD at 60° shoulder abduction (ICC = 0.57; bias = 0.05 cm; 95% LOA = -0.37–0.48 cm); (**C**) acromion-greater tuberosity (AGT) distance (ICC = 0.58; bias = -0.04 cm; 95% LOA = -0.89–0.81 cm); (**D**) supraspinatus tendon (SST) thickness in long axis (ICC = 0.60; bias = -0.01 cm; 95% LOA = -0.13–0.11 cm); (**E**) SST thickness in short axis (ICC = 0.64; bias = 0.00 cm, 95% LOA = -0.12–0.13 cm); (**F**) subacromial-subdeltoid bursa (SASDB) thickness in long axis #1 (ICC = 0.49; bias = -0.01 cm; 95% LOA = -0.09–0.07 cm); (**G**) SASDB thickness in long axis #2 (ICC = 0.56; bias = 0.01 cm; 95% LOA = -0.04–0.05 cm); (**H**) SASDB thickness in short axis (ICC = 0.54; bias = -0.01 cm; 95% LOA = -0.10–0.08 cm); (**I**) SASDB fluid thickness in long axis #1 (ICC = 0.43; bias = 0.01 cm; 95% LOA = -0.04–0.05 cm); (**J**) SASDB fluid thickness in long axis #2 (ICC = 0.58; bias = -0.02 cm; 95% LOA = -0.11–0.07 cm); and (**K**) SASDB fluid thickness in short axis (ICC = 0.53; bias = 0.01 cm; 95% LOA = -0.05–0.06 cm). Dotted lines represent the 95% LOA. ICC = intra-class correlation coefficient; LOA = limits of agreement.
